# Rapid Inhibition of Pyruvate Dehydrogenase: An Initiating Event in High Dietary Fat-Induced Loss of Metabolic Flexibility in the Heart

**DOI:** 10.1371/journal.pone.0077280

**Published:** 2013-10-07

**Authors:** Clair Crewe, Michael Kinter, Luke I. Szweda

**Affiliations:** 1 Free Radical Biology and Aging Research Program Oklahoma Medical Research Foundation, Oklahoma City, Oklahoma, United States of America; 2 Department of Biochemistry and Molecular Biology, University of Oklahoma Health Science Center, Oklahoma City, Oklahoma, United States of America; 3 Department of Geriatric Medicine, Reynolds Center on Aging, University of Oklahoma Health Science Center, Oklahoma City, Oklahoma, United States of America; University of Salento, Italy

## Abstract

Cardiac function depends on the ability to switch between fatty acid and glucose oxidation for energy production in response to changes in substrate availability and energetic stress. In obese and diabetic individuals, increased reliance on fatty acids and reduced metabolic flexibility are thought to contribute to the development of cardiovascular disease. Mechanisms by which cardiac mitochondria contribute to diet-induced metabolic inflexibility were investigated. Mice were fed a high fat or low fat diet for 1 d, 1 wk, and 20 wk. Cardiac mitochondria isolated from mice fed a high fat diet displayed a diminished ability to utilize the glycolytically derived substrate pyruvate. This response was rapid, occurring within the first day on the diet, and persisted for up to 20 wk. A selective increase in the expression of pyruvate dehydrogenase kinase 4 and inhibition of pyruvate dehydrogenase are responsible for the rapid suppression of pyruvate utilization. An important consequence is that pyruvate dehydrogenase is sensitized to inhibition when mitochondria respire in the presence of fatty acids. Additionally, increased expression of pyruvate dehydrogenase kinase 4 preceded any observed diet-induced reductions in the levels of glucose transporter type 4 and glycolytic enzymes and, as judged by Akt phosphorylation, insulin signaling. Importantly, diminished insulin signaling evident at 1 wk on the high fat diet did not occur in pyruvate dehydrogenase kinase 4 knockout mice. Dietary intervention leads to a rapid decline in pyruvate dehydrogenase kinase 4 levels and recovery of pyruvate dehydrogenase activity indicating an additional form of regulation. Finally, an overnight fast elicits a metabolic response similar to that induced by high dietary fat obscuring diet-induced metabolic changes. Thus, our data indicate that diet-induced inhibition of pyruvate dehydrogenase may be an initiating event in decreased oxidation of glucose and increased reliance of the heart on fatty acids for energy production.

## Introduction

Approximately 70% of the ATP produced in the heart is derived from the oxidation of fatty acids. The heart does, however, increase the utilization of glucose in response to enhanced availability, increased workload, and physiologic and pathophysiological stress. This metabolic flexibility is essential for cardiac function because: 1) Glucose relative to fatty acids produces more ATP per molecule of oxygen consumed and 2) Glycolytic intermediates are required for processes other than ATP production [1]. It is therefore noteworthy that obesity and diabetes result in diminished cardiac glucose oxidation and a heavy reliance on β-oxidation for energy production [2,3]. Chronic suppression of metabolic flexibility is widely believed to contribute to accompanying cardiovascular disease [4,5]. Importantly, a diet rich in fat promotes diminished cardiac glucose utilization prior to overt manifestations of obesity, diabetes, and cardiac dysfunction [6,7]. Therefore identifying early events and mechanisms that contribute to diet-induced metabolic inflexibility may offer promise for therapeutic intervention that reestablish proper metabolic balance.

The mitochondrial enzyme pyruvate dehydrogenase (PDH) is a key regulatory point in glucose oxidation, catalyzing the oxidative decarboxylation of pyruvate and formation of acetyl-CoA and NADH. PDH is stringently regulated, both allosterically and posttranslationally, based on energy demands as well as glucose and fatty acid availability. Reversible phosphorylation of PDH occurs on the α-chain of the E1 subunit, one of three subunits that comprise the PDH complex. Three phosphorylation sites exist (serine 293, 300, and 232 in mice). Phosphorylation of PDH inhibits enzyme activity and is catalyzed by four isoforms of pyruvate dehydrogenase kinase, three of which are expressed in the heart (PDK1, PDK2 and PDK4). Dephosphorylation and activation of PDH is catalyzed by two pyruvate dehydrogenase phosphatases (PDP1 and PDP2) [8-10]. Inhibition of PDH in the mouse heart by over-expression of PDK4 is sufficient to establish a metabolic profile similar to that seen in obesity, specifically increased β-oxidation and reduced glucose utilization [11,12]. Thus, altered regulation of cardiac PDH in response to high dietary fat has the potential to be an important contributor to the development of metabolic inflexibility.

Previous studies have demonstrated that mice fed a high fat diet exhibit increased expression of PDK4 mRNA [13,14] and/or protein [10,14-17] in cardiac and skeletal muscle. Diet-induced declines in the active fraction of PDH have also been reported [15,17,18]. Dietary durations of greater than one month were required, however, to observe these changes [7,10,15,16,18] suggestive of a downstream consequence rather than an initiating event in diminished glucose utilization. There are, however, reports that PDK4 [13,17] and PDH [17] exhibit changes within a few weeks of initiating a high fat diet. The lack of consensus on the time-frame of diet-induced alterations in certain measures of PDK4 expression or PDH activity may be due, in part, to variations in the fat content of experimental diets and/or animal models (i.e. strain, species) utilized. However, given the general similarities between many reported experimental approaches, disparate observations could be the result of differences in the methods for deducing changes in PDH activity and the length of time animals are fasted, a routinely employed protocol, prior to analysis. PDH activity is often evaluated in flash frozen heart tissue which has the benefit of capturing the activation state of PDH *in vivo*. Nevertheless, it is important to consider that PDH activity exhibits dynamic changes in response to shifting energy demands and substrate availability. Thus, depending on the method and/or time of sample preparation, the active fraction of PDH may not accurately reflect diet-induced changes in PDH function. Further confounding integration of reported findings, food deprivation is well known to elicit increases in PDK4 message, protein, and/or activity [16,18-21]. As such, fasting may conceal and/or diminish apparent effects of high dietary fat on PDK4 content and PDH activity. It has therefore been difficult to accurately assign a role for PDK4 upregulation and PDH inhibition in rapid diet-induced losses in glucose utilization and metabolic flexibility.

The regulation and activity of PDH under a variety of metabolic conditions were therefore evaluated using cardiac mitochondria isolated from mice fed high fat or control diets. A comprehensive approach was employed that included wild type and PDK4 knockout mice, evaluation of the phosphorylation status of each of the three sites on PDH, the development of a selected reaction monitoring proteomic method to quantify changes in all isoforms of PDK and PDP and each of the subunits of PDH, and select analyses of samples from fasted versus non-fasted experimental animals. The objectives of the current study were to assess the contribution of diet-induced alterations in PDK4 expression and PDH activity to initial declines in the capacity of mitochondria to utilize glycolytically derived pyruvate and distinguish between the effects of high dietary fat and fasting on these events. We therefore established: 1) The time-frame in which PDH is inhibited in response to high dietary fat; 2) Direct evidence that increased expression of PDK4 and/or other regulatory events are responsible for PDH inhibition; 3) The effects of fasting on apparent diet-induced changes in PDH activity and the content of PDK4; and 4) The impact of specific molecular alterations on mitochondrial substrate selection.

We demonstrate that a single day of high dietary fat induces appreciable reductions in pyruvate supported ADP-dependent respiration in isolated cardiac mitochondria as a result of a selective increase in the expression of PDK4 and inhibition of PDH. Inhibition of PDH is enhanced in the presence of fatty acids. Fasting elicits a convergent regulatory response concealing rapid metabolic changes provoked by high dietary fat. Furthermore, high fat diet-induced increases in PDK4 and inhibition of PDH precede declines in the level of glucose transporter 4 (GLUT4) and insulin stimulated Akt phosphorylation. In fact, reductions in cardiac insulin signaling evident after 1 wk of high dietary fat did not occur in PDK4 knockout mice. These findings provide evidence that PDK4 upregulation and PDH inhibition are a part of the immediate response of the heart to high dietary fat and may initiate diet-induced loss in glucose utilization.

## Experimental Procedures

### Mice and Diets

Male C57BL/6J mice were obtained from the Jackson Laboratory at 6 wk of age. PDK4^-/-^ mice were a gift from Dr. Robert A. Harris (Indiana University School of Medicine) [21]. At 8 wk of age mice were placed on a control (70% carbohydrate, 20% protein and 10% fat, by kcal) or high fat (20% carbohydrate, 20% protein and 60% fat, by kcal) diet (Research Diets Inc.) *ad libitum*. Mice exhibited increases in body weight as early as 1 wk on the high fat diet relative to mice maintained on a control diet (29.1 ± 4.4 g vs. 26.1 ± 1.6 g, *p* < 0.05, n = 12) with further increases over the time frame studied (44.3 ± 4.3 g vs. 30.8 ± 1.7 g at 20 wk, *p* < 0.001, n = 8). Mice were euthanized by cervical dislocation. All procedures were approved by the Oklahoma Medical Research Foundation Animal Care and Use Committee.

### Isolation of Cardiac Mitochondria

Immediately following euthanasia, hearts were excised and homogenized in ice-cold isolation buffer (10 mM MOPS, 1.0 mM EDTA, 210 mM mannitol, and 70 mM sucrose, pH 7.4) using a Potter-Elvehjem homogenizer. The homogenate was centrifuged at 550 x *g* for 5 min (4°C) and the supernatant was filtered through cheese cloth. The mitochondrial pellet was obtained by centrifugation of the supernatant at 10,000 x *g* for 10 min (4°C). Mitochondria were resuspended into homogenization buffer to a final concentration of 20.0 mg/mL. Protein determinations were made using the bicinchroninic acid (BCA) method (Pierce) with BSA as a standard.

### Analysis of Mitochondrial Respiratory Function

Mitochondria were diluted to 0.25 mg/mL in 10 mM MOPS, 210 mM mannitol, 70 mM sucrose, and 5.0 mM K_2_HPO_4_ at pH 7.4 (respiratory buffer) containing respiratory substrates (palmitoylcarnitine, pyruvate, malate) as indicated. State 3 respiration was initiated at 2.0 min by the addition of ADP at a final concentration of 0.25 mM. Rates of mitochondrial respiration were evaluated at room temperature using a Neofox oxygen chamber with a 175 uL volume (Instech Laboratories, Inc.).

### Evaluation of PDH Activity

Mitochondria were incubated under specified respiratory conditions at a concentration of 0.25 mg/ml for indicated times. Mitochondria were then diluted to 0.05 mg/mL in a buffer containing 25 mM MOPS and 0.05% Triton X-100 at pH 7.4. Solubilization of mitochondria with 0.05% Triton X-100 inhibits complex I of the respiratory chain preventing consumption of NADH. PDH activity was measured spectrophotometrically (Agilent, 8452A) as the rate of NAD^+^ reduction to NADH (340 nm, e = 6,200 M^-1^cm^-1^) upon addition of 2.5 mM pyruvate, 0.1 mM CoASH, 0.2 mM thiamine pyrophosphate, 1.0 mM NAD^+^, and 5.0 mM MgCl_2_ at pH 7.4.

### Western Blot Analysis

Mitochondria were suspended in loading buffer (106 mM Tris HCl, 141 mM Tris Base, 2.0% SDS, 10% sucrose, 100 mM DTT, 0.5 mM EDTA, 0.175 mM phenol red, 0.22 mM Brilliant Blue, at pH 8.5) with 20 mM NaF and protease inhibitor cocktail (Roche). Protein was then resolved on a NuPAGE 10% Bis-Tris gel (Life Technologies) and transferred to PVDF membrane (Bio-Rad). Anti-PDH E1α, anti-phospho-ser293, -ser300, and -ser232 were purchased from EMD Millipore, anti-pan AKT and anti-phospho-AKT(thr308) from Cell Signaling, and Hsp60 antibody from Santa Cruz Biotechnology. PDK4 antibody was a gift from Dr. Robert A. Harris (Indiana University School of Medicine). Primary antibody binding was visualized using secondary antibodies conjugated to horseradish peroxidase (Pierce) and SuperSignal West Pico Chemiluminescent Substrate (Thermo Scientific).

### Mass Spectrometry Analysis [22]

Quantitative proteomics was used to determine the levels of specific proteins. Mitochondrial protein was run 1.5 cm into a NuPAGE 12.5% SDS-PAGE gel (Criterion, Bio-Rad). The gel was then fixed and stained with GelCode Blue (Pierce). The entire lane was cut into ~1 mm^3^ pieces. The samples were washed, reduced with DTT, alkylated with iodoacetamide, and digested with trypsin. The peptides were extracted with 50% methanol/10% formic acid in water. The extract was dried and reconstituted in 1% acetic acid. The samples were analyzed using selected reaction monitoring with a triple quadrupole mass spectrometer (ThermoScientific TSQ Vantage) configured with a splitless capillary column HPLC system (Eksigent). The data were processed using the program Pinpoint (ThermoScientific), which aligned the various collision induced dissociation reactions monitored for each peptide and determined the chromatographic peak areas. The response for each protein was taken as the total response for all peptides monitored. Changes in the relative abundance of the proteins were determined by normalization to the BSA internal standard, with confirmation by normalization to the housekeeping proteins. For details of experimental protocol see Methods S1, Table S1, Figure S1, and Figure S2.

### Quantitative RT-PCR

Approximately 10 mg of heart tissue was snap frozen in liquid nitrogen. RNA was extracted using Tripure (Roche). A NanoDrop 2000 UV-Vis spectrophotometer (ThermoScientific) was used to determine RNA concentration. RNA (1.0 µg) was converted to cDNA (20 µl final volume) using the QuantiTect Reverse Transcription kit (Qiagen). Quantitative PCR was performed on a CFX96 thermocycler (BioRad) with reactions consisting of 1.0 µL cDNA, 125 nM final concentration of each primer, and iQ SYBR Green Supermix (BioRad) in a total volume of 20 µL. Ct values for each sample were averaged from technical duplicates. Relative transcript expression ratios and statistics were generated using REST2009 software (Pfaffl pauls). The transcript levels for target genes were normalized to three reference genes (Gapdh, Sdha, and Hspcb) determined to be stable between dietary conditions using geNorm analysis (Vandesompele-pauls).

### Cardiac Insulin Signaling Assay

Mice received an intraperitoneal injection (0.05 U/g) of human insulin (NovoLog, Novo Nordisk Inc.). Hearts were harvested 5 min post-injection and snap-frozen in liquid nitrogen. Samples were then prepared for Western blot analysis.

### Statistics

Data is presented as mean ± SEM. Statistical analyses were preformed using the 2-tailed Student’s *t*-test and the Bonferroni correction for multiple comparisons with *p* values denoted throughout the text as: * < 0.05; ** < 0.01; and *** < 0.001.

## Results

### The Rate of Pyruvate Utilization is Reduced in Cardiac Mitochondria Isolated from Mice Fed a High Fat Diet for as Little as 1 Day

**Figure 1:**
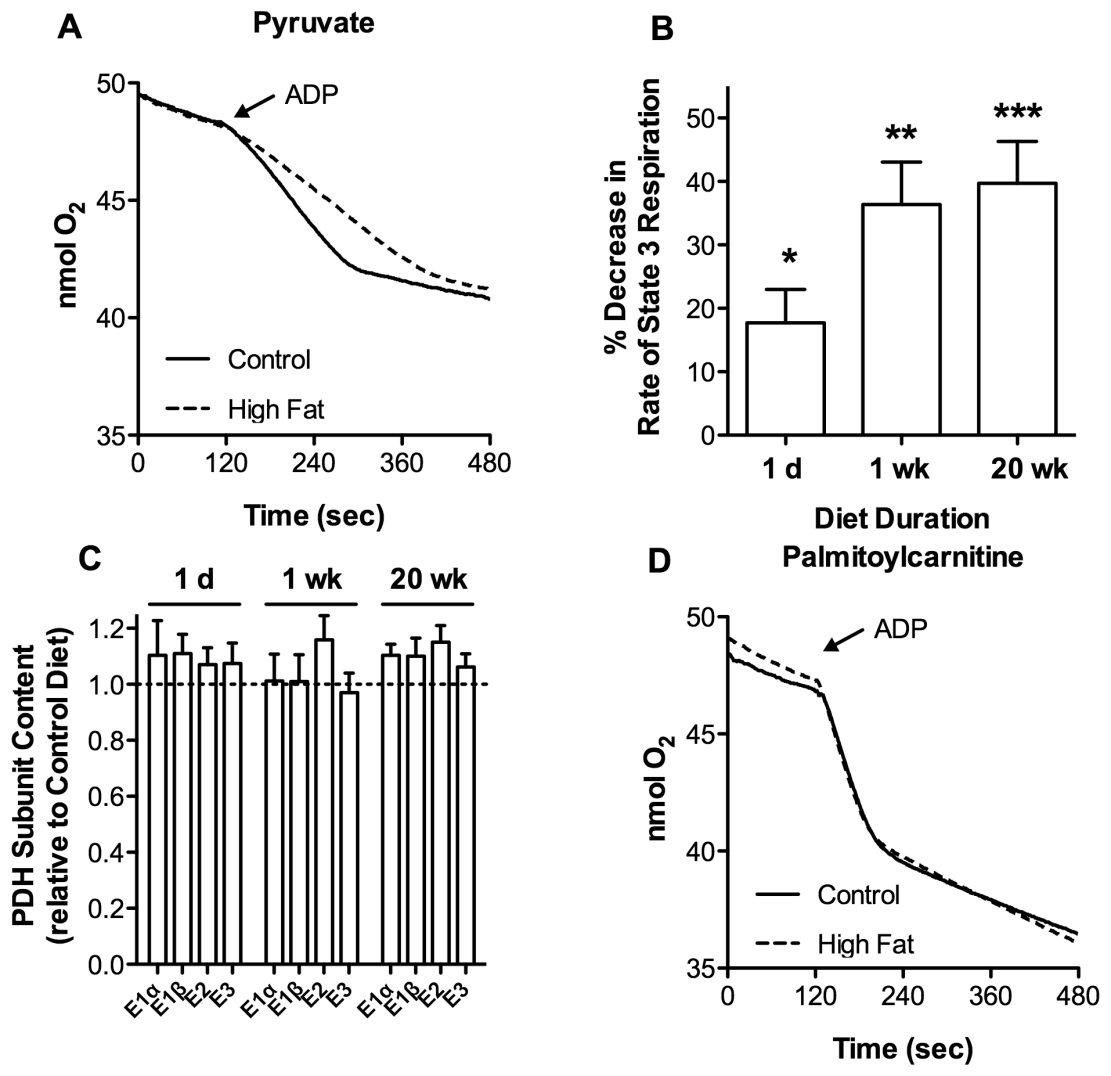
High Dietary Fat Reduces the Rate of Pyruvate Supported Respiration in Isolated Cardiac Mitochondria. C57BL/6 mice were fed a control or high fat diet for 1 d to 20 wk. Cardiac mitochondria were then isolated and incubated at 0.25 mg/mL with 25 µM palmitoylcarnitine and 1.0 mM malate or 100 µM pyruvate and 1.0 mM malate as respiratory substrates. State 3 respiration was initiated by the addition of 0.25 mM ADP at 2.0 min. Representative oxygen consumption traces for mitochondria isolated from mice fed control or high fat diet for 20 wk using **A**. pyruvate or **D**. palmitoylcarnitine. **B**. Percentage decrease in the rate of pyruvate-supported ADP-dependent (state 3) respiration relative to controls for diet durations indicated (n = 5, 11, and 9 for 1 d, 1wk, and 20 wk). C. Mass spectrometric quantification of the protein levels of each PDH subunit (n = 5). All data are presented as the mean ± SEM with *p* values: * < 0.05; ** < 0.01; and *** < 0.001.

We sought evidence for decreased mitochondrial pyruvate utilization as a contributor to loss in metabolic flexibility induced by high dietary fat and the time frame under which defined changes occur. C57BL/6J mice were fed a high fat or control diet (60% kcal versus 10% kcal from fat) for up to 20 wk. Cardiac mitochondria were isolated at various dietary durations and respiratory function was measured with either pyruvate or palmitoylcarnitine as the oxidative substrate. With pyruvate, mitochondria isolated from mice on the high fat diet (20 wk) exhibited a ~40% reduction in state 3 respiration relative to mitochondria from control animals (Figure 1A and Table 1). A reduced rate of state 3 respiration was evident within 1 d of high dietary fat with near maximal inhibition at 1 wk (Figure 1B). Thus, diminished mitochondrial pyruvate utilization occurs rapidly in response to high dietary fat and persists for up to 20 wk. Inhibition of state 3 respiration could, however, be overcome by increasing the concentration of pyruvate (Table 1) indicating that the mechanism responsible is not likely diminished content of proteins that support pyruvate linked respiration. Consistent with this interpretation, mass spectrometric analysis found no change in the protein content for each of the PDH subunits in whole heart homogenate from mice fed high dietary fat for up to 20 wk (Figure 1C). With palmitoylcarnitine, the rate of ADP-dependent (state 3) respiration was unaffected by 20 wk of high dietary fat (Figure 1D and Table 1). In addition, there was no change in the rate of ADP-independent (state 4) respiration. Thus, mitochondrial respiratory capacity and integrity appear unaltered by high dietary fat.

**Table 1:** Effects of High Dietary Fat on Cardiac Mitochondrial Respiratory Activity.

**Substrate:**	Pyruvate (100 µM)		Pyruvate (1.0 mM)		Palmitoylcarnitine (25 µM)	
	State 3	State 4	State 3	State 4	State 3	State 4
**Low Fat:**	89.0 ± 5.1***	13.7 ± 1.6	100.1 ± 7.0	18.1 ± 1.5	201.8 ± 24.2	28.4 ± 2.8
**High Fat:**	53.6 ± 5.9***	12.7 ± 1.7	90.2 ± 8.1	15.4 ± 0.7	217.5 ± 22.1	34.8 ± 3.3

Cardiac mitochondria were isolated from mice fed control or high fat diet for 20 wk. Mitochondria were incubated with 100 µM pyruvate, 1.0 mM pyruvate, or 25 µM palmitoylcarnitine in the presence of 1.0 mM malate. State 3 respiration was initiated by addition of 0.25 mM ADP at 2.0 min. The rates (nmol O/min/mg) of ADP-dependent (state 3) and ADP-independent (state 4) respiration are represented as the mean ± SEM with *p* value: *** < 0.001 (n = 9).

### PDH Activity is Diminished in Respiring Mitochondria Following 1 Day of High Fat Feeding

**Figure 2:**
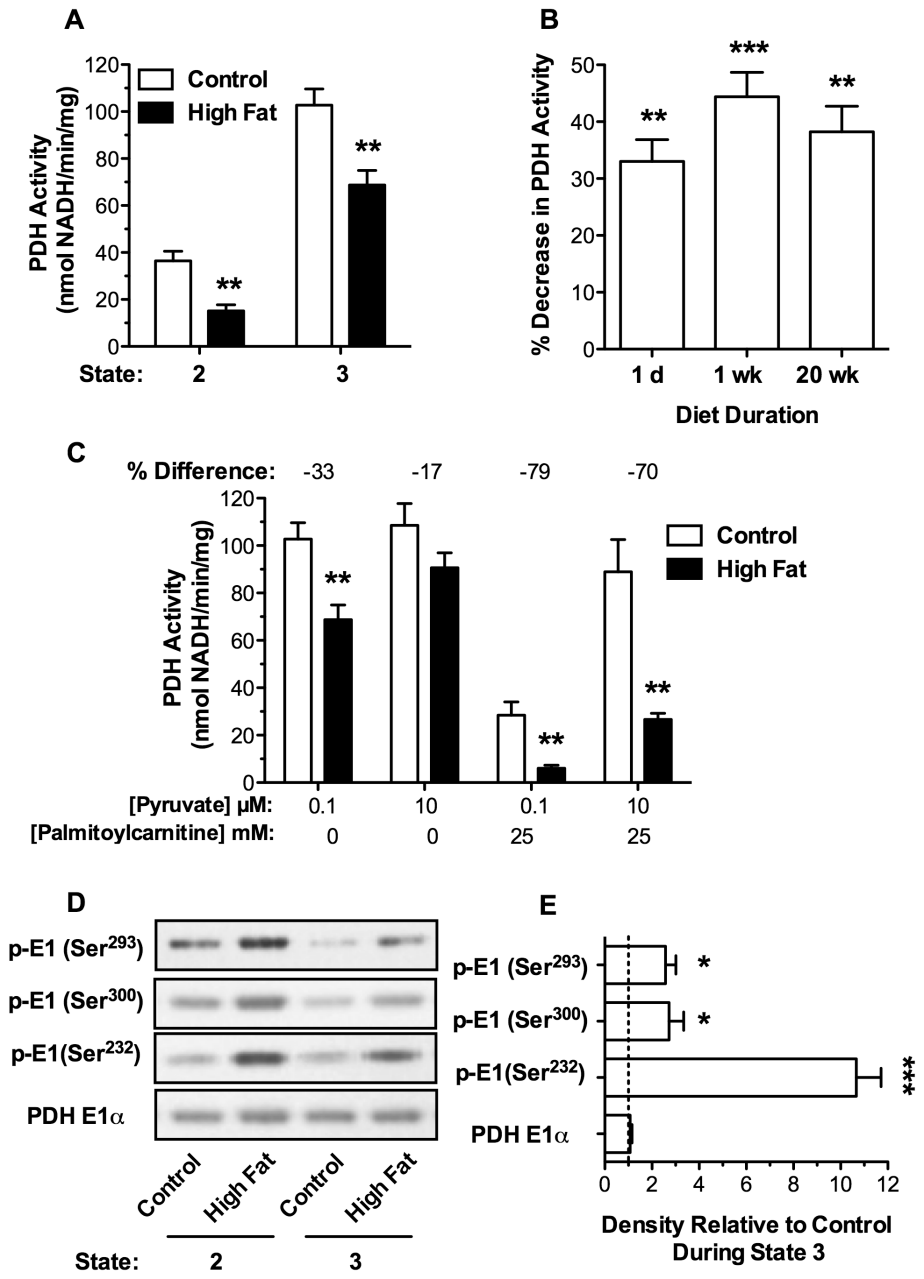
Pyruvate Dehydrogenase Activity is Diminished and Phosphorylation Enhanced in Respiring Cardiac Mitochondria from Mice Fed High Dietary Fat. Cardiac mitochondria (0.25 mg/mL) were incubated with 100 µM pyruvate and 1.0 mM malate. At 2.0 min, state 3 respiration was initiated by the addition of 0.25 mM ADP. **A**. PDH activity at 1.5 min, during state 2 respiration, and at 4.5 min, during state 3 respiration, in cardiac mitochondria from mice fed control or high fat diet for 1 d (n = 5). **B**. PDH activity at 4.5 min, during state 3 respiration, for cardiac mitochondria from mice fed a control or high fat diet for indicated times. Data are presented as the % decrease relative to controls (n = 5, 6, and 6 for 1 d, 1 wk, and 20 wk). **C**. Mitochondria were incubated with 100 µM or 10 mM pyruvate and 1.0 mM malate in the presence or absence of 25 µM palmitoylcarnitine. At 2.0 min, ADP was added (0.25 mM) to initiate state 3 respiration and PDH activity was assayed at 4.5 min (n = 5). **D**. Western blot analysis (representative of n = 5) of phosphorylation status for each site on the PDH E1α subunit during state 2 and 3 respiration in cardiac mitochondria isolated from mice on indicated diets for 1 d. E. Relative phosphorylation status during state 3 respiration in cardiac mitochondria isolated from mice fed a control (arbitrary unit 1) versus high fat diet for 1 d quantified by densitometric analysis of the Western blots (n = 5). All data are presented as the mean ± SEM with *p* values: * < 0.05; ** < 0.01; and *** < 0.001.

To evaluate mechanisms responsible for diminished pyruvate-supported respiration, the dynamic regulation of PDH was assessed as a function of mitochondrial respiratory state. Mitochondria isolated from mice on either diet exhibited a significant increase in PDH activity during state 3 respiration when demand for ATP synthesis is high. However, absolute PDH activity was significantly depressed at each respiratory state for cardiac mitochondria isolated from mice fed a high fat relative to control diet for 1 d (Figure 2A). The loss in PDH activity during state 3 respiration remained relatively constant with increasing diet duration up to 20 wk (Figure 2B).

### High Dietary Fat Sensitizes PDH to Inhibition in the Presence of Fatty Acids

To further assess the effects of high dietary fat on PDH activity, mitochondria were incubated in the presence of pyruvate and fatty acid, arguably a more physiological condition. Mitochondria from hearts of mice fed a high fat or control diet for 1 d were incubated, under state 3 respiratory conditions, with 100 µM or 10 mM pyruvate in the absence or presence of palmitoylcarnitine. As shown in Figure 2C, high pyruvate concentrations (10 mM), in the absence of palmitoylcarnitine resulted in enhanced PDH activity in both dietary groups. Under these conditions the difference in PDH activity was diminished from 33% to 17% (Figure 2C). These results are consistent with our observation that increasing the concentration of pyruvate relieved high fat diet-induced inhibition of state 3 respiration (Table 1). Importantly, when mitochondria respired with pyruvate in the presence of palmitoylcarnitine, the relative difference in PDH activity between the two diets increased (79%, *p* < 0.01) and increasing the pyruvate concentration from 100 µM to 1 mM (not shown) or 10 mM (Figure 2C) had little effect. Similar results were obtained for mice fed the respective diets for 1 and 20 wk (not shown). These data indicate that the PDH complex in mitochondria from hearts of mice fed a high fat diet is more sensitive to inhibition in the presence of fatty acids.

### PDH Phosphorylation is Enhanced in Respiring Mitochondria Isolated From Mice Fed a Diet High in Fat

PDH can be inhibited by phosphorylation of 3 serine residues on the E1α subunit. Reflective of reduced enzyme activity, phosphorylation of PDH was elevated during states 2 and 3 respiration in mitochondria isolated from mice fed a high fat relative to control diet (Figure 2D and E). Increased phosphorylation of PDH was evident at all 3 phosphorylation sites under both state 2 and 3 respiratory conditions (Figure 2D and E). However, ser^232^ exhibited the greatest fold increase in phosphorylation during state 3 respiration. Similar phosphorylation patterns were observed in mitochondria isolated from mice fed the respective diets for 1 and 20 wk (data not shown). Enhanced phosphorylation is therefore likely responsible for diet-induced reductions in PDH activity (Figure 2A and B). The contribution of increased PDK and/or reduced PDP expression was investigated.

### PDK4 Expression is Selectively Increased in Response to High Dietary Fat

**Figure 3:**
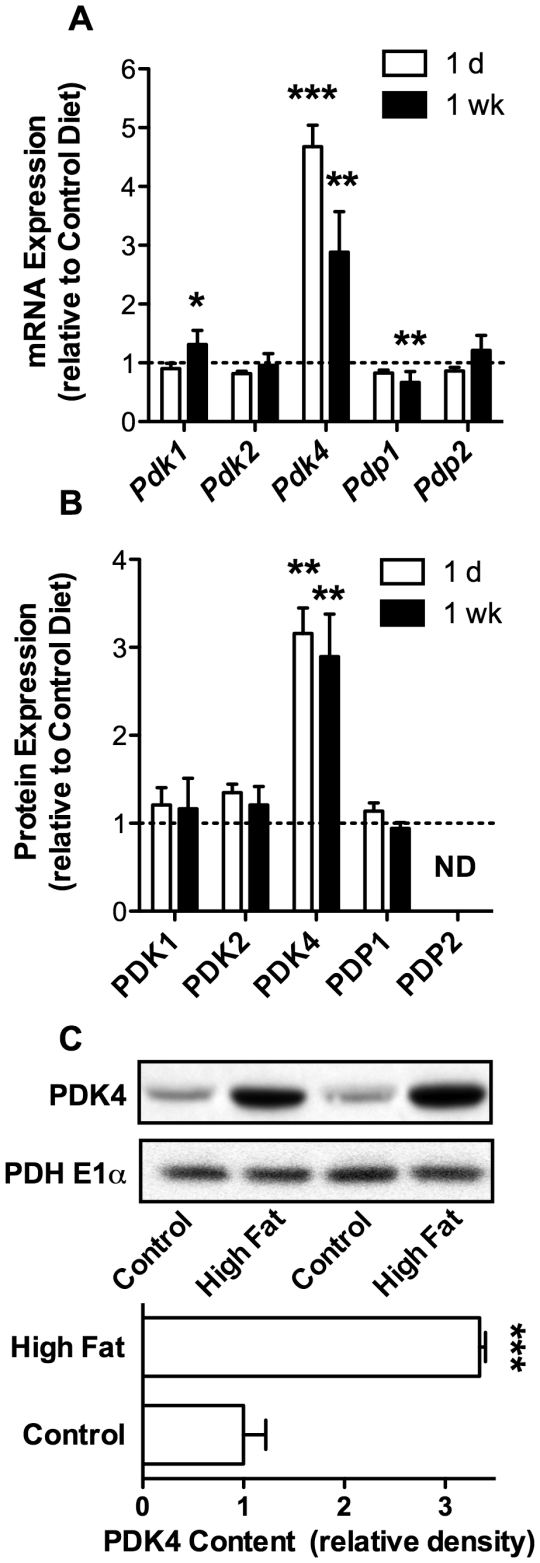
High Dietary Fat Drives a Selective Increase in the Expression of PDK4 in the Heart. mRNA and protein content of PDK and PDP isoforms were measured in hearts of mice fed either the control or high fat diet for 1 d or 1 wk. **A**. mRNA expression determined by qRT-PCR (n = 5 and 9 for 1 d and 1 wk). **B**. Protein content measured by quantitative mass spectrometry (n = 5 and 6 for 1 d and 1 wk). **C**. Expression of PDK4 in isolated cardiac mitochondria quantified by densitometric analysis of Western blots (n = 5 for 1 wk). All data are presented as the mean ± SEM with *p* values: ** < 0.01; and *** < 0.001.

mRNA and protein levels of each PDK and PDP isoform in heart tissue from mice fed high fat or control diets were quantified. PDK4 expression showed the most dramatic increase in response to the high fat diet. PDK4 mRNA increased 4.6- and 2.8-fold at 1 d and 1 wk of high fat feeding, respectively (Figure 3A). PDK4 protein levels increased approximately 3-fold at each dietary duration (Figure 3B), as determined by mass spectrometry. The increase in PDK4 protein content was confirmed by Western blot analysis (Figure 3C). PDK1 and PDP1 mRNA increased and decreased ~30%, respectively (Figure 3A). Despite these changes at the transcript level, no statistical change was detected at the protein level by mass spectrometry (Figure 3B). PDK2 did not exhibit diet-induced alterations in mRNA or protein content (Figure 3A and B). These results indicate that PDK4 expression increases rapidly in response to high dietary fat and is likely the primary mechanism by which PDH is inhibited during state 3 respiration.

### High-Dietary Fat Alters the Kinetics of PDH Activation and Inhibition in Response to Energy Demand

**Figure 4:**
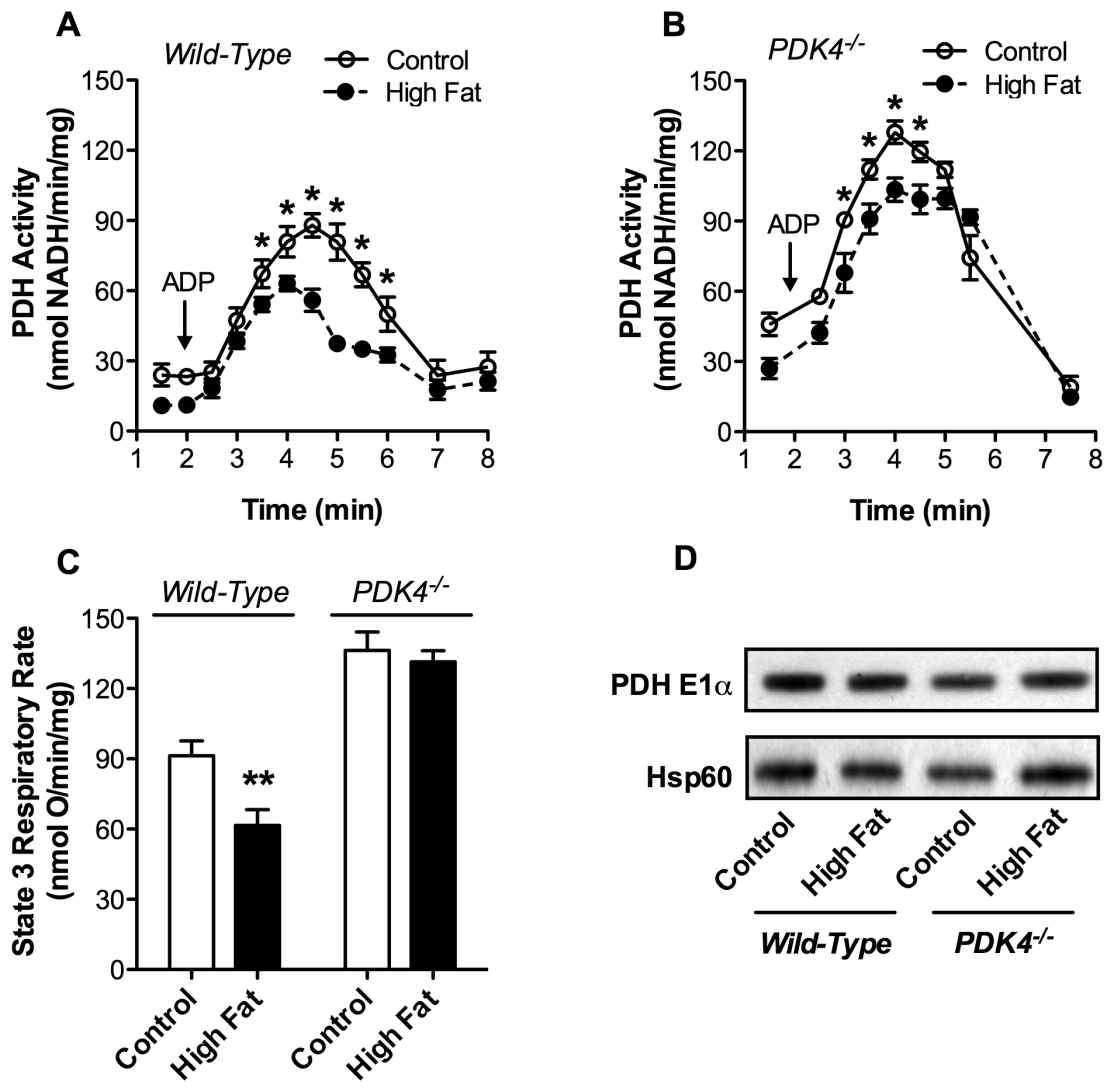
High Dietary Fat Alters the Kinetics of PDH Inhibition in Wild-type but not PDK4-/- Mice. Cardiac mitochondria (0.25 mg/mL) from mice fed control or high fat diets for 1 wk were incubated with 100 µM pyruvate and 1.0 mM malate. At 2.0 min, state 3 respiration was initiated by the addition of 0.25 mM ADP. PDH activity was assayed at indicated time points for **A**. wild-type mice (n = 5) and **B**. PDK4^-/-^ mice (n = 5). **C**. Oxygen consumption traces for mitochondria respiring with pyruvate were recorded. The rate of state 3 respiration was calculated for wild-type mice (n = 11) and PDK4^-/-^ mice (n = 5). **D**. The relative levels of PDH E1α and Hsp60 as a loading control were assessed in wild-type and PDK4^-/-^ mice fed control or high fat diet by Western blot analysis. All data are presented as the mean ± SEM with *p* values: * < 0.05 and ** < 0.01.

Relative to other PDK isoforms, PDK4 is more sensitive to activation by the products of fatty acid and pyruvate oxidation [8,10,23]. Mitochondria isolated from hearts of mice fed high fat relative to control diet might therefore be expected to exhibit differential alterations in PDH activity during the course of state 3 respiration. The kinetics of activation and subsequent inhibition of PDH were assessed in isolated mitochondria respiring on pyruvate. Activation of the enzyme occurs upon addition of ADP in mitochondria isolated from mice maintained on either diet (Figure 4A). PDH in cardiac mitochondria from mice fed a high fat relative to control diet did not, however, reach the same level of activation. This appears due to early initiation of PDH inhibition, despite a reduced rate and therefore prolonged duration of state 3 respiration (Figure 4A and C). In fact, at 4.5 min, when PDH activity begins to decline in mitochondria isolated from mice fed a high fat diet, only 21.4% ± 5.3 of the ADP had been converted to ATP relative to 31.3% ± 5.4 for mitochondria from control animals (p < 0.05).

### Diet-Induced Inhibition of PDH and Pyruvate Supported Respiration is Reduced in PDK4 Knockout Mice

PDK4 knockout mice were used to test the role of increased PDK4 expression in reducing PDH and mitochondrial respiratory activity in response to high dietary fat. As shown in Figure 4A and 4B, high fat diet-induced inhibition of PDH was significantly less in PDK4^-/-^ mice relative to wild type animals (16% vs 36%). More importantly, cardiac mitochondria isolated from PDK4^-/-^ mice fed a high fat diet for 1 wk did not display the rapid inhibition of PDH during state 3 respiration (Figure 4B) observed in wild type mice (Figure 4A). No change in mitochondrial respiratory activity was observed (Figure 4C). Compensation by other kinases was not evident in PDK4^-/-^ mice. In response to high dietary fat, PDK1 expression increased 17% (p < 0.01) with no changes in PDK2, PDP1 or PDP2 mRNA levels (data not shown), a result similar to that observed for wild type mice (Figure 3A). These data demonstrate that increased expression of PDK4 is the major mechanism responsible for inhibition of cardiac PDH in response to high dietary fat. The importance of PDK4 in the regulation of PDH is underscored by a 50% increase in PDH activity (128.0 ± 4.8 vs. 87.9 ± 5.0 nmol NADH/min/mg, *p* < 0.01) and the rate of state 3 respiration (136.3 ± 7.9 vs. 91.3 ± 6.1 nmol O/min/mg, *p* < 0.001) in PDK4 knockout relative to wild-type mice fed the control diet (Figure 4A-C) despite no change in PDH E1α protein content (Figure 4D).

### PDH Inhibition is Reversible When Mice are Removed From the High Fat Diet

**Figure 5:**
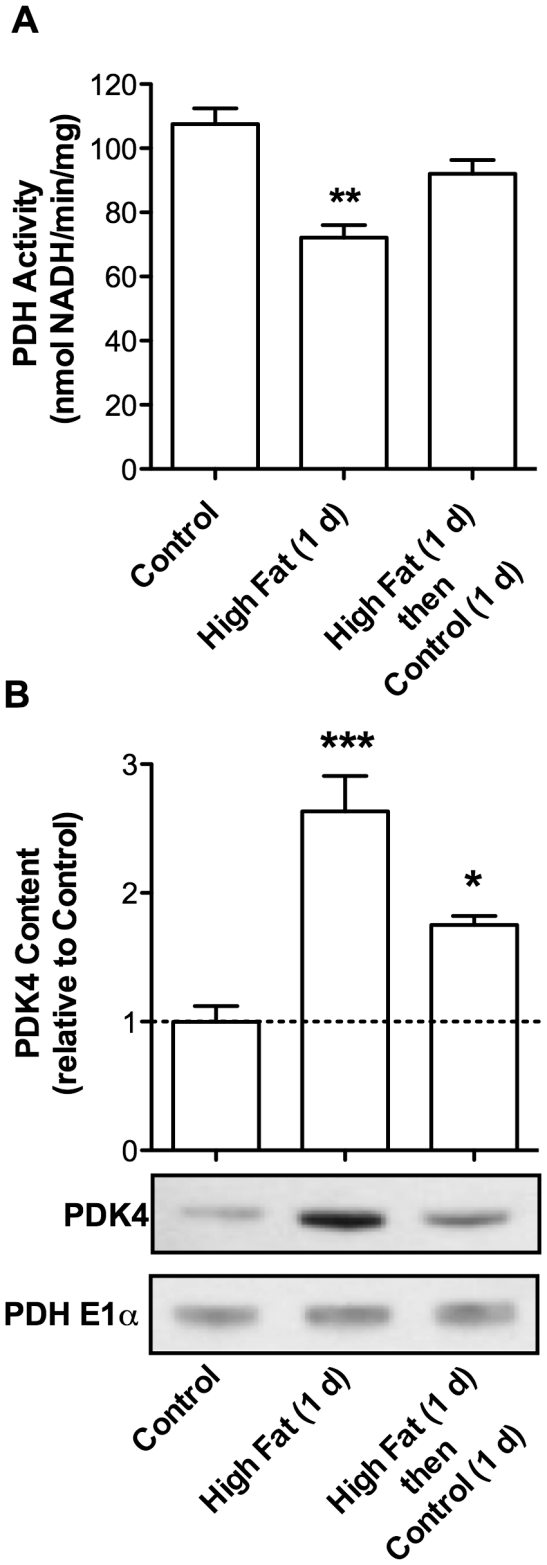
PDH Inhibition is Relieved and PDK4 Content Reduced Upon Removal of Mice from the High Fat Diet. C57BL/6 mice were fed either a control diet for 1 d, a high fat diet for 1 d, or a high fat diet for 1 d followed by a control diet for 1 d. **A**. Cardiac mitochondria were incubated with 100 µM pyruvate and 1.0 mM malate. State 3 respiration was initiated by the addition of ADP (0.25 mM). PDH activity was assayed during state 3 respiration (n = 3). **B**. Western blot analysis (representative of n = 3) and quantitative mass spectrometry (n = 3) were used to determine PDK4 protein levels in isolated cardiac mitochondria. All data are presented as the mean ± SEM with *p* values: * < 0.05; ** < 0.01; and *** < 0.001.

To determine if metabolic changes observed after 1 d of high dietary fat are rapidly reversible, mice were randomly assigned to 1 of 3 diet regimes: 1) Control diet; 2) High fat diet for 1 d; and 3) High fat diet for 1 d followed by control diet for 1 d. In mice fed a high fat diet for 1 d, PDH activity was suppressed by ~30% relative to controls (Figure 5A) accompanied by 2.6-fold increase in PDK4 expression (Figure 5B). Dietary intervention for a single day partially relieved inhibition of PDH and reduced the level of PDK4 (Figure 5). The rapid reduction in PDK4 content upon the return of animals to a control diet suggests protein degradation as an additional form of regulation.

### Rapid PDH Inhibition Induced by High Dietary Fat is Concealed When Mice are Fasted Prior to Analysis

**Figure 6:**
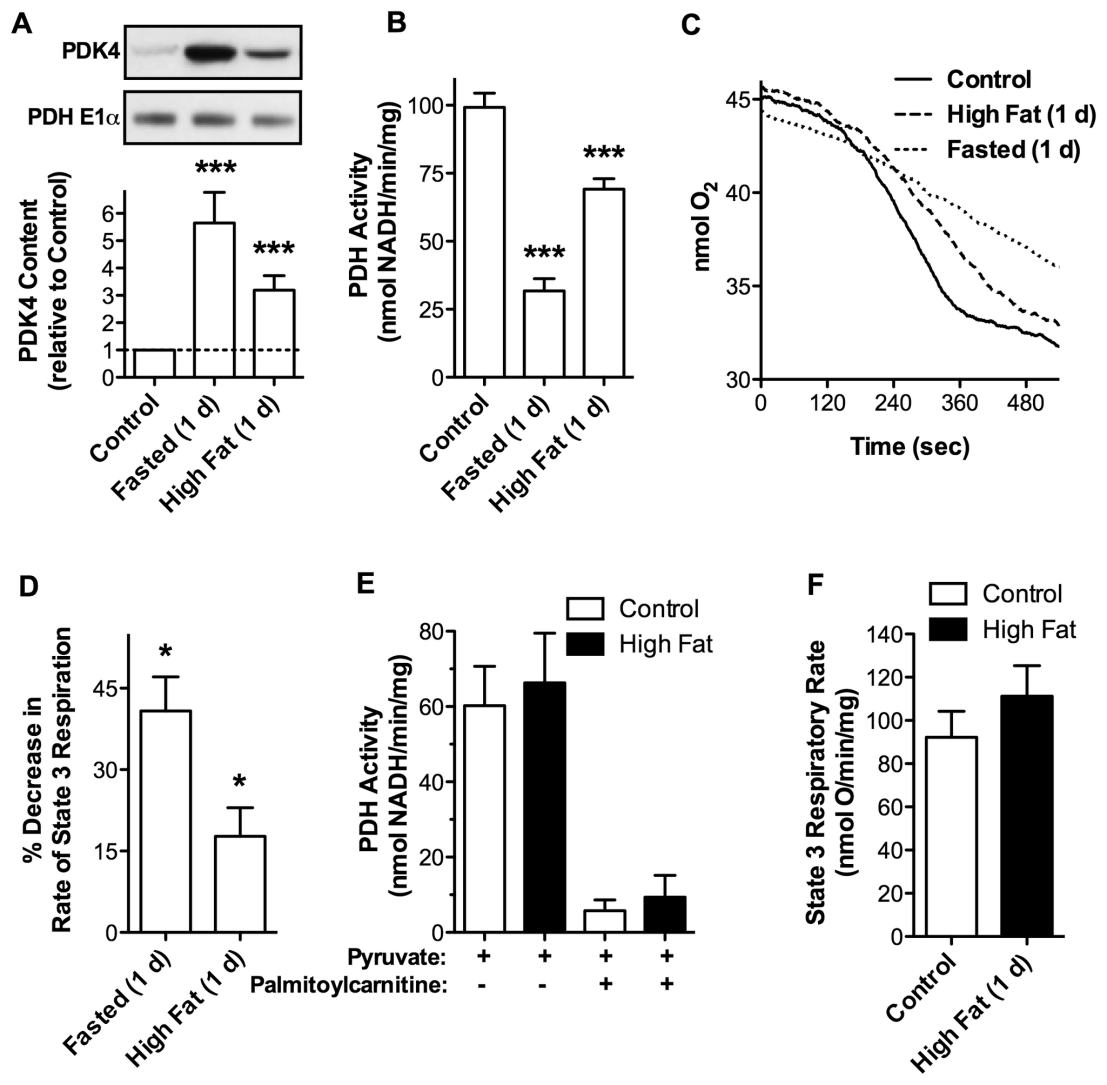
Fasting Conceals Rapid Diet-Induced Inhibition of PDH. Mice were fasted for 24 h or fed control or high fat diets for 1 d. Mitochondria were incubated with 100 µM pyruvate and 1.0 mM malate. State 3 respiration was initiated by addition of 0.25 mM ADP. **A**. PDK4 levels in isolated cardiac mitochondria assessed by Western blot analysis (representative of n = 5) and mass spectrometry (n = 5). **B**. PDH activity was assayed at 4.5 min during state 3 respiration with 100 µM pyruvate and 1.0 mM malate in cardiac mitochondria from mice fed the indicated diets (n = 5). **C**. Representative oxygen consumption traces for cardiac mitochondria from mice that were fasted or maintained on control or high fat diets for 1 d. **D**. Percent decrease in state 3 respiratory rate for each dietary condition relative to respective controls (n = 5). **E**. Mice were fed control or high fat diets for 2 d. All mice were fasted overnight (12 h) before sacrifice. PDH activity was assayed at 4.5 min during state 3 respiration with 100 µM pyruvate and 1.0 mM malate in the presence or absence of 25 µM palmitoylcarnitine. **F**. Rate of pyruvate supported state 3 respiration of cardiac mitochondria from mice fasted overnight (12 h) after indicated diets. All data are presented as the mean ± SEM with *p* values: * < 0.05 and *** < 0.001.

It is well known that cardiac PDK4 expression increases during starvation [19,20]. We determined whether an increase in PDK4 induced by starvation (24 h) had effects on PDH activity and mitochondrial respiratory function similar to those observed in response to a high fat diet (24 h). Cardiac mitochondria isolated from mice deprived of food for 24 h exhibited a dramatic 5-fold increase in PDK4 protein content (Figure 6A) and a 65% reduction in PDH activity relative to controls (Figure 6B). These changes were accompanied by a 40% reduction in the rate of state 3 respiration with pyruvate as a respiratory substrate (Figure 6C and D). Similar metabolic changes were observed at 1 d on the high fat diet, although to a lesser magnitude (Figure 6A-D). These observations raise the possibility that an overnight fast (12 h), a traditional experimental protocol, might obscure the rapid nature by which high dietary fat elicits an increase in PDK4 expression and PDH inhibition. Mice were fed a high fat or control diet for 2 d. All mice were then fasted overnight (12 h) prior to analysis. No differences were detected in PDH activity (Figure 6E), pyruvate supported respiration (Figure 6F), or PDK4 levels (not shown) between animals fed a high fat versus control diet. When compared to mice fed a control diet that were not fasted prior to analysis, PDK4 levels increased (2.87 + 0.69 fold versus 2.32 + 0.93 fold), and PDH was inhibited (Figure 6B and E) to similar extents in mice fed a high fat or control diet followed by a 12 h fast. Thus, the traditional protocol of fasting animals prior to assessing metabolic effects of diet fully masks rapid PDK4-mediated inhibition of PDH arising from high dietary fat.

### Diet-Induced Increases in PDK4 Content and Inhibition of PDH Precede Changes in the Expression of Glycolytic Enzymes and GLUT4 and Cardiac Insulin Signaling

**Figure 7:**
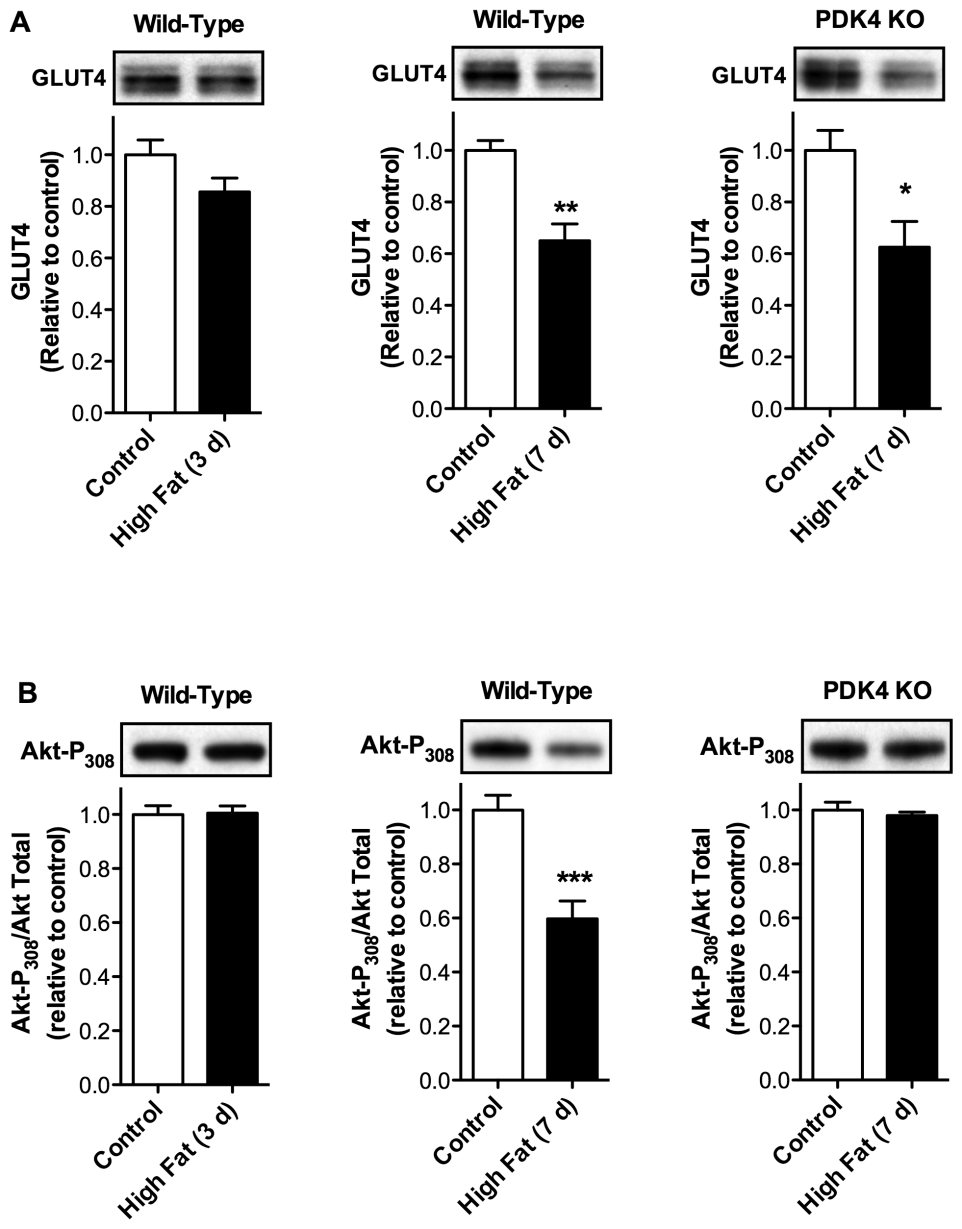
PDK4-Upregulation Occurs Prior to Reductions in GLUT4 Expression and Insulin-Stimulated Akt Phosphorylation. Mice were placed on a high fat or control diet for 3 d or 1 wk. Hearts were then processed and **A**. GLUT4 expression was determined by Western blot analysis and quantified by densitometric analysis (n = 5) and **B**. Cardiac insulin signaling was assessed as insulin-stimulated Akt phosphorylation, quantified by densitometric analysis (n = 5), and represented relative to total Akt. As indicated, wild type or PDK4 knockout mice were utilized. All data are presented as the mean ± SEM with *p* values: * < 0.05 and *** < 0.001.

To determine the temporal relationship between diet-induced changes in PDH inhibition arising from upregulation of PDK4 and other measures of glycolytic capacity, we evaluated the effects of short-term high fat diet on cardiac levels of glycolytic enzymes and the glucose transporter, GLUT4, as well as cardiac insulin signaling. Mass spectrometric analysis found no alterations in the level of glycolytic enzymes in heart tissue from mice fed a high fat diet for 1 wk (Table 2). Conversely, there was a significant reduction in GLUT4 protein at 1 wk of high fat feeding; however, this effect was not observed at 3 d (Figure 7A). Similarly, cardiac insulin signaling, as measured by insulin-stimulated Akt phosphorylation, was significantly reduced at 1 wk but not 3 d of high dietary fat (Figure 7B). Interestingly, PDK4 knockout mice did not exhibit a loss of insulin signaling after 1 wk of high fat feeding (Figure 7B). In contrast, GLUT4 levels declined even in the absence of PDK4 (Figure 7A). Therefore, increased expression of PDK4 and inhibition of PDH precede diet-induced declines in GLUT4 content and insulin-stimulated Akt phosphorylation. In addition, diet-induced increases in PDK4 appear to play a role in loss of cardiac insulin signaling.

**Table 2:** Effects of High Dietary Fat on Cardiac Content of Glycolytic Enzymes.

**Enzyme**	**Protein Content (high fat relative to control)**
Hexokinase-1	0.93 ± 0.06
Phosphoglucose Isomerase 1	0.98 ± 0.04
Phosphofructokinase-1	1.02 ± 0.06
Aldolase A	1.12 ± 0.08
Triose Phosphate Isomerase	1.18 ± 0.05
Glceraldehyde 3-Phosphate Dehydrogenase	1.00 ± 0.01
Enolase 3	1.10 ± 0.05
Pyruvate Kinase	0.98 ± 0.06
Lactate Dehydrogenase A (1)	1.06 ± 0.08
Lactate Dehydrogenase B (2)	1.12 ± 0.08

Mice were placed on a high fat or control diet for 1 wk. Heart extracts were then subjected to mass spectrometric analysis for expression of glycolytic enzymes. Data is represented as the mean ± SEM of the ratio of protein abundance in hearts from mice fed high fat relative to control diet (n = 5). No statistical differences were observed.

## Discussion

We have made the novel discovery that, within 1 d of a high fat diet, mice exhibit a decrease in the capacity to utilize glycolytically derived pyruvate in isolated cardiac mitochondria. A selective increase in the expression of PDK4 and inhibition of PDH are responsible. High dietary fat did not induce inhibition of PDH in PDK4^-/-^ mice. Furthermore increased expression of PDK4 precedes diet-induced reductions in cardiac insulin signaling, as judged by insulin stimulated Akt phosphorylation, and the content of the glucose transporter GLUT4, or any changes in the levels of glycolytic enzymes measured. Increases in PDK4 content and/or a decline in the fraction of active PDH have previously been reported to occur with high dietary fat, however, these changes were observed at dietary durations on the order of weeks or greater [7,10,15-18], often after diet-induced loss of glucose transport or insulin signaling [7,10,15,16,18]. The rapid effects of high dietary fat on PDK4 content and PDH activity may go undetected because fasting, often performed prior to metabolic analyses, induces a similar increase in PDK4 levels or activity [16,18-21] and, as we have shown, masks the specific contribution of a high fat diet. Thus, we have identified a mechanism that, based on the rapidity with which it occurs, may initiate declines in metabolic flexibility arising from high dietary fat, opening new lines of investigation.

Increased delivery and utilization of fatty acids for energy production will inherently lead to inhibition of PDH. This is, in large part, due to inhibition of PDH and activation of various isoforms of PDK by acetyl-CoA and NADH produced during the oxidation of fatty acids [8,10,24-26]. A question that emerges is why then selectively and rapidly increase the expression PDK4, in response to high dietary fat? PDK4, relative to the other isoforms, is more sensitive to activation by NADH and less sensitive to inhibition by pyruvate and ADP [8,10,15,23]. Thus, a selective increase in PDK4 expression favors phosphorylation and inhibition of PDH under conditions where PDH would normally be in the active state. Consistent with the properties of PDK4 and the enhanced expression of this isoform with high fat feeding, initiation of PDH inhibition was observed early in pyruvate-supported state 3 respiration in mitochondria isolated from mice fed a high fat relative to control diet. Increased expression of PDK4 enhances the degree of PDH inhibition in the presence of fatty acid, a condition that cannot be overcome by elevated pyruvate concentrations. As a result, the rapid molecular events triggered by high dietary fat ensure stringent use of fatty acids by the heart, irrespective of glucose and pyruvate availability.

From an evolutionary perspective, organisms experienced dramatic changes in food availability and macronutrient composition in response to seasonal, regional, and environmental changes. Mechanisms for rapidly shifting cardiac metabolism to reflect both substrate availability and appropriate partitioning of fatty acids and glucose to various tissues would have provided a survival advantage. During starvation, PDK4-dependent inhibition of PDH and increased reliance on fatty acids for energy production spares glucose for use by tissues more dependent on glycolysis than the heart [19-21]. In fact, PDK4^-/-^ relative to wild-type mice exhibit diminished fasting blood glucose [21]. In contrast, glucose is not limiting with high fat diets. Glycolytically derived pyruvate is, however, an important anaplerotic substrate for replenishment of Krebs cycle intermediates [27-29]. During high fat feeding, concentrations of pyruvate may be limited due to allosteric inhibition of glycolysis by products of fatty acid oxidation [8,10,24-26]. This effect would be exacerbated during longer diet durations when cardiac insulin resistance develops and glucose uptake is reduced [3,6,7]. A selective increase in PDK4 and stringent inhibition of PDH would restrict conversion of pyruvate to acetyl-CoA and may therefore spare limited pyruvate for anaplerosis ultimately providing support for increased flux through β-oxidation to ensure the use of available fatty acids.

Despite the potential advantages of such a response, long term high fat feeding leads to obesity and diabetes [3,30], major risk factors for cardiovascular disease and heart failure [2,4,5]. A common link between obesity, diabetes, and cardiac dysfunction is atherosclerosis and accompanying ischemic events. In this scenario, glucose utilization is favorable and diminished metabolic flexibility is detrimental for cardiovascular recovery and function [1,3]. In fact, malonyl-CoA decarboxylase knockout (MCD^-/-^) mice that exhibit diminished cardiac β-oxidation do not undergo diet-induced loss of cardiac insulin sensitivity and glucose utilization and are protected from cardiac ischemia and reperfusion injury [31,32]. Similarly, whole body PDK4 knockout and peroxisome proliferator-activated receptor α (PPARα) functional knockout mice, also characterized by enhanced glucose oxidation and suppressed β-oxidation, are protected from high fat diet-induced reductions in glucose tolerance and insulin sensitivity [33,34]. In addition, we demonstrate that PDK4 knockout mice do not exhibit a loss in cardiac insulin signaling, as measured by insulin stimulated Akt phosphorylation, following 1 wk of high dietary fat. Given the relatively recent advent of chronic high caloric diets in the background of diminished physical activity, diabetes and atherosclerosis are not likely to have posed evolutionary constraints. Therefore, rapid induction of PDK4 and inhibition of PDH may be a regulatory mechanism to ensure use of available fatty acids that, with increasing dietary durations, exerts deleterious effects reflective of a lifestyle that has little evolutionary traction.

The findings of the current study necessitate future identification of mechanism(s) by which PDK4 expression is rapidly upregulated and contributes to loss of insulin sensitivity. Several transcription factors, including PPARα, FOXO-1, ERRα, E2F1, and PGC-1α, are known to influence the expression of PDK4 [10,17,19,35]. What remains to be determined is which transcription factors predominate at different dietary durations, particularly the acute phase, and in response to varying dietary composition. In addition, certain transcription factors may indirectly affect PDK4 expression by altering levels of components of related metabolic pathways, such as β-oxidation. It is therefore important to identify transcription factors that bind to the PDK4 promoter region in response to changing dietary conditions. Moreover, the rapid decline in PDK4 content with dietary intervention suggests degradation as an additional form of regulation. A potential mechanism by which increased expression of PDK4 precipitates loss of insulin signaling is through metabolically driven changes in the redox environment [36]. Mitochondrial H_2_O_2_ production is higher when metabolizing fatty acids relative to pyruvate [37-39]. . Treatment of cells with H_2_O_2_ has been shown to reduce insulin signaling [40]. In addition, targeted consumption of mitochondria derived H_2_O_2_ prevents diet-induced reductions in insulin-stimulated Akt phosphorylation within the heart [41]. Nevertheless, definitive evidence that oxidative modifications to specific components of insulin signaling occur and are responsible for observed diet-induced functional declines is lacking. Finally, evidence indicates that increases in PDK4 content and inhibition of PDH are not required for declines in cardiac GLUT4 content arising from high dietary fat. Future studies must therefore define the relative contributions of diminished GLUT4 to defects in glucose uptake and utilization and mechanism(s) responsible. Overall, elucidation of interrelated and complex molecular events that initiate and drive diet-induced metabolic inflexibility is required if interventions that go beyond countering downstream deficits and specifically target underlying causes are to be developed.

## Supporting Information

Methods S1 **Details of experimental protocol for Mass Spectrometry Analysis.**
(DOCX)

Figure S1 **Sample preparation by short run gel electrophoresis.** An aliquot of a whole heart homogenate containing 60µg protein (typically about 100µL) is mixed with 50µL 10% SDS and an internal standard solution containing 8pmol BSA and 1pmol chicken lysozyme. The sample is heated at 70°C for 15min to ensure complete solubilization. The proteins are then precipitated with 1mL acetone overnight at -20°C. The protein pellet is collected, reconstituted in 60µL sample loading buffer, and a 20µL aliquout (20µg) run into a 12.5% SDS-Page gel (BioRad Criterion) at 150V for 15min. The gel is washed, fixed, and stained with Coomassie blue (GelCode Blue, Pierce).
(TIF)

Figure S2 **Representative LC-tandem mass spectrometry data for the measurement of Pdha1, Pdhb, and Pdk4.** The raw LC-tandem MS data were processed using the Pinpoint program (ThermoScientific). These images are taken directly from that program. Two peptides were monitored for each protein and are shown in the first two columns. For each peptide, the figures contain chromatographic peaks for the respective peptides for a set of analyses of 5 fasted animals, 5 control animals, and a heart homogenate pool that is used for quality control. For each chromatographic peak, the different colors represent the different fragmentation reactions that are monitored for that peptide. The y-axis is the relative abundance. The x-axis is labeled with the respective raw data filename. The final column shows the total signal for each protein, plotted as the mean ± standard deviation. The y-axis is the relative abundance. The x-axis is labeled for the three types of samples; animals fasted for 1day (n=5), animals feeding ad lib with a control diet (n=5), and a standard sample from a mouse heart homogenate pool (n=1) that is used for quality control.
(TIF)

Table S1 **The selected reaction monitoring descriptors used to detect Pdha, Pdhb, Pdk1, Pdk2, and Pdk4 within a multiplexed quantitative assay.** This assay included a group of 31 proteins including the Krebs cycle enzymes, housekeeping proteins, an internal standard, and selected other mitochondrial enzymes. The C[160] designates an alkylated cysteine. All analyses are in the positive ion mode with Q1 and Q3 resolution set to 0.7Da.
(DOCX)

## References

[1] Stanley WC, Recchia FA, Lopaschuk GD (2005) Myocardial substrate metabolism in the normal and failing heart. Physiol Rev 85: 1093-1129. doi:10.1152/physrev.00006.2004. PubMed: 15987803. 15987803

[2] Abel ED, Litwin SE, Sweeney G (2008) Cardiac remodeling in obesity. Physiol Rev 88: 389-419. doi:10.1152/physrev.00017.2007. PubMed: 18391168. 18391168 PMC2915933

[3] Lopaschuk GD, Ussher JR, Folmes CDL, Jaswal JS, Stanley WC (2010) Myocardial Fatty Acid Metabolism in Health and Disease. Physiol Rev 90: 207-258. doi:10.1152/physrev.00015.2009. PubMed: 20086077. 20086077

[4] Hubert HB, Feinleib M, McNamara PM, Castelli WP (1983) Obesity as an independent risk factor for cardiovascular disease: a 26-year follow-up of participants in the Framingham Heart Study. Circulation 67: 968-977. doi:10.1161/01.CIR.67.5.968. PubMed: 6219830. 6219830

[5] Kenchaiah S, Evans JC, Levy D, Wilson PW, Benjamin EJ et al. (2002) Obesity and the risk of heart failure. N Engl J Med 347: 305-313. doi:10.1056/NEJMoa020245. PubMed: 12151467. 12151467

[6] Park SY, Cho YR, Kim HJ, Higashimori T, Danton C et al. (2005) Unraveling the temporal pattern of diet-induced insulin resistance in individual organs and cardiac dysfunction in C57BL/6 mice. Diabetes 54: 3530-3540. doi:10.2337/diabetes.54.12.3530. PubMed: 16306372. 16306372

[7] Wright JJ, Kim J, Buchanan J, Boudina S, Sena S et al. (2009) Mechanisms for increased myocardial fatty acid utilization following short-term high-fat feeding. Cardiovasc Res 82: 351-360. PubMed: 19147655. 19147655 10.1093/cvr/cvp017 PMC2675931

[8] Patel MS, Korotchkina LG (2006) Regulation of the pyruvate dehydrogenase complex. Biochem Soc Trans 34: 217-222. doi:10.1042/BST20060217. PubMed: 16545080. 16545080

[9] Rardin MJ, Wiley SE, Naviaux RK, Murphy AN, Dixon JE (2009) Monitoring phosphorylation of the pyruvate dehydrogenase complex. Anal Biochem 389: 157-164. doi:10.1016/j.ab.2009.03.040. PubMed: 19341700. 19341700 PMC2713743

[10] Sugden MC, Holness MJ (2006) Mechanisms underlying regulation of the expression and activities of the mammalian pyruvate dehydrogenase kinases. Arch Physiol Biochem 112: 139-149. doi:10.1080/13813450600935263. PubMed: 17132539. 17132539

[11] Chambers KT, Leone TC, Sambandam N, Kovacs A, Wagg CS et al. (2011) Chronic inhibition of pyruvate dehydrogenase in heart triggers an adaptive metabolic response. J Biol Chem 286: 11155-11162. doi:10.1074/jbc.M110.217349. PubMed: 21321124. 21321124 PMC3064169

[12] Zhao G, Jeoung NH, Burgess SC, Rosaaen-Stowe KA, Inagaki T et al. (2008) Overexpression of pyruvate dehydrogenase kinase 4 in heart perturbs metabolism and exacerbates calcineurin-induced cardiomyopathy. Am J Physiol Heart Circ Physiol 294: H936-H943. doi:10.1152/ajpheart.00870.2007. PubMed: 18083902. 18083902

[13] Wilson CR, Tran MK, Salazar KL, Young ME, Taegtmeyer H (2007) Western diet, but not high fat diet, causes derangements of fatty acid metabolism and contractile dysfunction in the heart of Wistar rats. Biochem J 406: 457-467. doi:10.1042/BJ20070392. PubMed: 17550347. 17550347 PMC2049036

[14] Rinnankoski-Tuikka R, Silvennoinen M, Torvinen S, Hulmi JJ, Lehti M et al. (2012) Effects of high-fat diet and physical activity on pyruvate dehydrogenase kinase-4 in mouse skeletal muscle. Nutr Metab (Lond) 9: 53. doi:10.1186/1743-7075-9-53. PubMed: 22682013. 22682013 PMC3407034

[15] Holness MJ, Kraus A, Harris RA, Sugden MC (2000) Targeted upregulation of pyruvate dehydrogenase kinase (PDK)-4 in slow-twitch skeletal muscle underlies the stable modification of the regulatory characteristics of PDK induced by high-fat feeding. Diabetes 49: 775-781. doi:10.2337/diabetes.49.5.775. PubMed: 10905486. 10905486

[16] Holness MJ, Smith ND, Bulmer K, Hopkins T, Gibbons GF et al. (2002) Evaluation of the role of peroxisome-proliferator-activated receptor alpha in the regulation of cardiac pyruvate dehydrogenase kinase 4 protein expression in response to starvation, high-fat feeding and hyperthyroidism. Biochem J 364: 687-694. PubMed: 12049632. 12049632 10.1042/BJ20011841 PMC1222617

[17] Zhang L, Mori J, Wagg C, Lopaschuk GD (2012) Activating cardiac E2F1 induces up-regulation of pyruvate dehydrogenase kinase 4 in mice on a short term of high fat feeding. FEBS Lett 586: 996-1003. doi:10.1016/j.febslet.2012.02.027. PubMed: 22569253. 22569253

[18] Orfali KA, Fryer LGD, Holness MJ, Sugden MC (1993) Long-term regulation of pyruvate dehydrogenase kinase by high-fat feeding. Experiments in vivo and in cultured cardiomyocytes. FEBS Lett 336: 501-505. doi:10.1016/0014-5793(93)80864-Q. PubMed: 8282119. 8282119

[19] Wu PF, Peters JM, Harris RA (2001) Adaptive increase in pyruvate dehydrogenase kinase 4 during starvation is mediated by peroxisome proliferator-activated receptor alpha. Biochem Biophys Res Commun 287: 391-396. doi:10.1006/bbrc.2001.5608. PubMed: 11554740. 11554740

[20] Wu PF, Sato J, Zhao Y, Jaskiewicz J, Popov KM et al. (1998) Starvation and diabetes increase the amount of pyruvate dehydrogenase kinase isoenzyme 4 in rat heart. Biochem J 329: 197-201. PubMed: 9405294. 9405294 10.1042/bj3290197 PMC1219032

[21] Jeoung NH, Wu P, Joshi MA, Jaskiewicz J, Bock CB et al. (2006) Role of pyruvate dehydrogenase kinase isoenzyme 4 (PDHK4) in glucose homoeostasis during starvation. Biochem J 397: 417-425. doi:10.1042/BJ20060125. PubMed: 16606348. 16606348 PMC1533314

[22] Kinter CS, Lundie JM, Patel H, Rindler PM, Szweda LI et al. (2012) A quantitative proteomic profile of the Nrf2-mediated antioxidant response of macrophages to oxidized LDL determined by selected reaction monitoring. PLOS ONE 7: e50016. doi:10.1371/journal.pone.0050016. PubMed: 23166812. 23166812 PMC3500347

[23] Bowker-Kinley MM, Davis WI, Wu PF, Harris RA, Popov KM (1998) Evidence for existence of tissue-specific regulation of the mammalian pyruvate dehydrogenase complex. Biochem J 329: 191-196. PubMed: 9405293. 9405293 10.1042/bj3290191 PMC1219031

[24] Hue L, Taegtmeyer H (2009) The Randle cycle revisited: a new head for an old hat. Am J Physiol Endocrinol Metab 297: E578-E591. doi:10.1152/ajpendo.00093.2009. PubMed: 19531645. 19531645 PMC2739696

[25] Randle PJ (1998) Regulatory interactions between lipids and carbohydrates: the glucose fatty acid cycle after 35 years. Diabetes/Metab Rev 14: 263-283. doi:10.1002/(SICI)1099-0895(199812)14:4. PubMed: 10095997. 10095997

[26] Randle PJ, Garland PB, Hales CN, Newsholme EA (1963) The glucose fatty-acid cycle. Its role in insulin sensitivity and the metabolic disturbances of diabetes mellitus. Lancet 1: 785-789. PubMed: 13990765. 13990765 10.1016/s0140-6736(63)91500-9

[27] Des Rosiers C, Labarthe F, Lloyd SG, Chatham JC (2011) Cardiac anaplerosis in health and disease: food for thought. Cardiovasc Res 90: 210-219. doi:10.1093/cvr/cvr055. PubMed: 21398307. 21398307 PMC3078802

[28] Gibala MJ, Young ME, Taegtmeyer H (2000) Anaplerosis of the citric acid cycle: role in energy metabolism of heart and skeletal muscle. Acta Physiol Scand 168: 657-665. doi:10.1046/j.1365-201x.2000.00717.x. PubMed: 10759602. 10759602

[29] Russell RR, Taegtmeyer H (1991) Pyruvate carboxylation prevents the decline in contractile function of rat hearts oxidizing acetoacetate. Am J Physiol 261: H1756-H1762. PubMed: 1750532. 1750532 10.1152/ajpheart.1991.261.6.H1756

[30] Muoio DM, Neufer PD (2012) Lipid-induced mitochondrial stress and insulin action in muscle. Cell Metab 15: 595-605. doi:10.1016/j.cmet.2012.04.010. PubMed: 22560212. 22560212 PMC3348508

[31] Ussher JR, Koves TR, Jaswal JS, Zhang L, Ilkayeva O et al. (2009) Insulin-stimulated cardiac glucose oxidation is increased in high-fat diet-induced obese mice lacking malonyl CoA decarboxylase. Diabetes 58: 1766-1775. doi:10.2337/db09-0011. PubMed: 19478144. 19478144 PMC2712785

[32] Ussher JR, Wang W, Gandhi M, Keung W, Samokhvalov V et al. (2012) Stimulation of glucose oxidation protects against acute myocardial infarction and reperfusion injury. Cardiovasc Res 94: 359-369. doi:10.1093/cvr/cvs129. PubMed: 22436846. 22436846

[33] Guerre-Millo M, Rouault C, Poulain P, André J, Poitout V et al. (2001) PPAR-alpha-null mice are protected from high-fat diet-induced insulin resistance. Diabetes 50: 2809-2814. doi:10.2337/diabetes.50.12.2809. PubMed: 11723064. 11723064

[34] Hwang B, Jeoung NH, Harris RA (2009) Pyruvate dehydrogenase kinase isoenzyme 4 (PDHK4) deficiency attenuates the long-term negative effects of a high-saturated fat diet. Biochem J 423: 243-252. doi:10.1042/BJ20090390. PubMed: 19627255. 19627255

[35] Wende AR, Huss JM, Schaeffer PJ, Giguère V, Kelly DP (2005) PGC-1alpha coactivates PDK4 gene expression via the orphan nuclear receptor ERRalpha: a mechanism for transcriptional control of muscle glucose metabolism. Mol Cell Biol 25: 10684-10694. doi:10.1128/MCB.25.24.10684-10694.2005. PubMed: 16314495. 16314495 PMC1316952

[36] Fisher-Wellman KH, Neufer PD (2012) Linking mitochondrial bioenergetics to insulin resistance via redox biology. Trends Endocrinol Metab 23: 142-153. doi:10.1016/j.tem.2011.12.008. PubMed: 22305519. 22305519 PMC3313496

[37] Rindler PM, Plafker SM, Szweda LI, Kinter M (2013) High Dietary Fat Selectively Increases Catalase Expression within Cardiac Mitochondria. J Biol Chem 288: 1979-1990. doi:10.1074/jbc.M112.412890. PubMed: 23204527. 23204527 PMC3548505

[38] Seifert EL, Estey C, Xuan JY, Harper ME (2010) Electron transport chain-dependent and -independent mechanisms of mitochondrial H2O2 emission during long-chain fatty acid oxidation. J Biol Chem 285: 5748-5758. doi:10.1074/jbc.M109.026203. PubMed: 20032466. 20032466 PMC2820802

[39] St-Pierre J, Buckingham JA, Roebuck SJ, Brand MD (2002) Topology of superoxide production from different sites in the mitochondrial electron transport chain. J Biol Chem 277: 44784-44790. doi:10.1074/jbc.M207217200. PubMed: 12237311. 12237311

[40] Iwakami S, Misu H, Takeda T, Sugimori M, Matsugo S et al. (2011) Concentration-dependent dual effects of hydrogen peroxide on insulin signal transduction in H4IIEC hepatocytes. PLOS ONE 6: e27401. doi:10.1371/journal.pone.0027401. PubMed: 22102892. 22102892 PMC3216925

[41] Anderson EJ, Lustig ME, Boyle KE, Woodlief TL, Kane DA et al. (2009) Mitochondrial H2O2 emission and cellular redox state link excess fat intake to insulin resistance in both rodents and humans. J Clin Invest, 119: 573–81. PubMed: 19188683. 19188683 10.1172/JCI37048 PMC2648700

