# Supplementary material for: Rapid Inhibition of Pyruvate Dehydrogenase: An Initiating Event in High Dietary Fat-Induced Loss of Metabolic Flexibility in the Heart
Source: PLoS One. 2013 Oct 7;8(10):e77280. doi: 10.1371/journal.pone.0077280 (PMC3792029; doi:10.1371/journal.pone.0077280)
Supplement: Methods S1 — Details of experimental protocol for Mass Spectrometry Analysis. (DOCX) [file pone.0077280.s001.docx]

**METHODS S1.**

**Details of experimental protocol for Mass Spectrometry Analysis**

The proteins in each sample were quantified by LC-tandem mass spectrometry using selected reaction monitoring (SRM) (Reference S1). This quantitative proteomics method is based on measuring peptides, formed by complete tryptic digestion of protein homogenates, as surrogates for the intact protein. The more abundant the protein is in a sample, the more abundant the peptides are in the digest, and the stronger the signals are in the LC-tandem mass spectrometry experiment. For these analyses, 20 µg protein (either whole heart homogenate or isolated mitochondria) are prepared by a short-run electrophoresis step that immobilized the protein is a small section of polyacrylamide gel before an in-gel tryptic digestion (Figure S1). The gel is fixed, stained, and inspected to assure the integrity of the samples and equal loading. Each lane is cut as a single sample, reduced with DTT, alkylated with iodoacetamide, and digested with 1 µg trypsin.

The LC-tandem mass spectrometry system is programmed to detect approximately 65 peptides, from 31 proteins, in a single experiment. The mass spectrometry system is a TSQ-Vantage from ThermoScientific with a splitless nanoflow HPLC system with autoinjector from Eksigent. A 10 cm C18 column (Phenomenex Jupiter) packed in a fused silica electrospray tip (New Objective) was used. 5 µL to 10 µL Volumes of the samples were injected. The column was eluted at 160nL/min with a linear gradient of CH_3_CN in water with 0.1% formic acid (3% CH3CN to 63% CH_3_CN in 30 min). Each peptide was monitored in a 5min time window based on its retention time. Two peptides were measured for each protein and multiple collision induced dissociation reactions are monitored for each peptide. In this assay, Pdha1, Pdhb, Pdk1, Pdk2, and Pdk4 were measured in a group of Krebs cycle proteins, several housekeeping proteins (Hspd1 and Vdac1), and an internal standard protein (BSA). The SRM conditions for Pdha1, Pdhb, Pdk1, Pdk2, and Pdk4 are given in Table S1.

The data are analyzed by integrating the chromatographic peak area for each peptide. The abundance of a given protein is determined as the total area of the two peptides. Changes in expression are seen in multiple ways, including based on the raw signal intensity (Figure S2), based on ratios obtained by normalization to the two housekeeping proteins, and based on normalization to the BSA internal standard. The normalization to the BSA internal standard was also used to express the protein amounts as pmol/mg protein. This calculation represents an estimate of protein amount using the best flyer approach proposed by Aebersold and co-workers (Reference S2).

**REFERENCES**

S1. Kinter CS, Lundie JM, Patel H, Rindler PM, Szweda LI, et al. (2012) A quantitative proteomic profile of the Nrf2-mediated antioxidant response of macrophages to oxidized LDL determined by selected reaction monitoring. PLoS ONE 7: e50016.

S2. Ludwig C, Claassen M, Schmid A, Aebersold R (2012) Estimation of absolute protein quantities of unlabeled samples by selected reaction monitoring mass spectrometry. *Mol Cell Proteomics* 11: 10.1074/mcp.M111.013987, 1–16.
