# Supplementary figures and images for: Rapid Inhibition of Pyruvate Dehydrogenase: An Initiating Event in High Dietary Fat-Induced Loss of Metabolic Flexibility in the Heart
Source: PLoS One. 2013 Oct 7;8(10):e77280. doi: 10.1371/journal.pone.0077280 (PMC3792029; doi:10.1371/journal.pone.0077280)

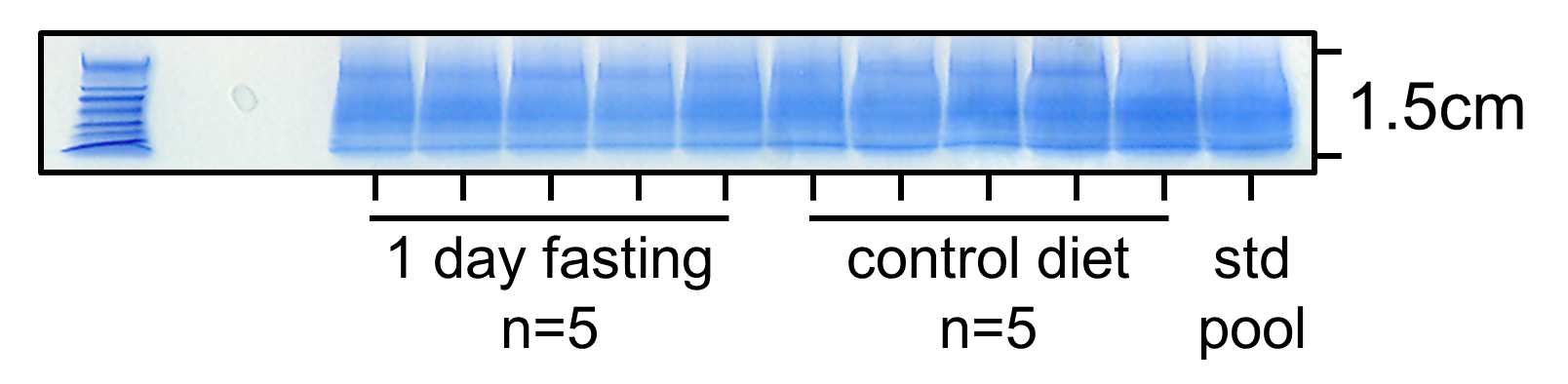

Supplement: Figure S1 — Sample preparation by short run gel electrophoresis. An aliquot of a whole heart homogenate containing 60µg protein (typically about 100µL) is mixed with 50µL 10% SDS and an internal standard solution containing 8pmol BSA and 1pmol chicken lysozyme. The sample is heated at 70°C for 15min to ensure complete solubilization. The proteins are then precipitated with 1mL acetone overnight at -20°C. The protein pellet is collected, reconstituted in 60µL sample loading buffer, and a 20µL aliquout (20µg) run into a 12.5% SDS-Page gel (BioRad Criterion) at 150V for 15min. The gel is washed, fixed, and stained with Coomassie blue (GelCode Blue, Pierce). (TIF) [file pone.0077280.s002.tif]

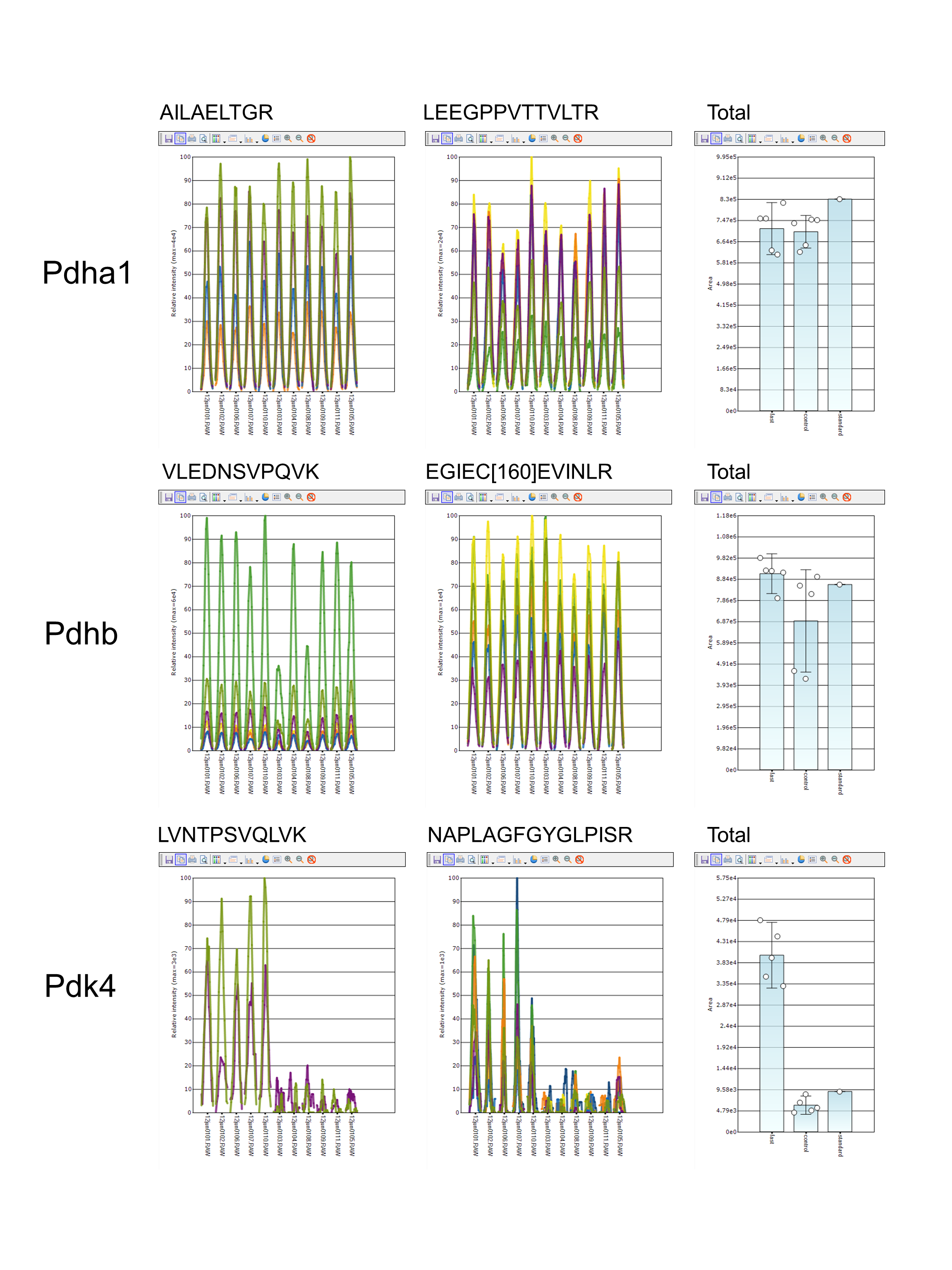

Supplement: Figure S2 — Representative LC-tandem mass spectrometry data for the measurement of Pdha1, Pdhb, and Pdk4. The raw LC-tandem MS data were processed using the Pinpoint program (ThermoScientific). These images are taken directly from that program. Two peptides were monitored for each protein and are shown in the first two columns. For each peptide, the figures contain chromatographic peaks for the respective peptides for a set of analyses of 5 fasted animals, 5 control animals, and a heart homogenate pool that is used for quality control. For each chromatographic peak, the different colors represent the different fragmentation reactions that are monitored for that peptide. The y-axis is the relative abundance. The x-axis is labeled with the respective raw data filename. The final column shows the total signal for each protein, plotted as the mean ± standard deviation. The y-axis is the relative abundance. The x-axis is labeled for the three types of samples; animals fasted for 1day (n=5), animals feeding ad lib with a control diet (n=5), and a standard sample from a mouse heart homogenate pool (n=1) that is used for quality control. (TIF) [file pone.0077280.s003.tif]
