# Supplementary material for: Rapid Inhibition of Pyruvate Dehydrogenase: An Initiating Event in High Dietary Fat-Induced Loss of Metabolic Flexibility in the Heart
Source: PLoS One. 2013 Oct 7;8(10):e77280. doi: 10.1371/journal.pone.0077280 (PMC3792029; doi:10.1371/journal.pone.0077280)
Supplement: Table S1 — The selected reaction monitoring descriptors used to detect Pdha, Pdhb, Pdk1, Pdk2, and Pdk4 within a multiplexed quantitative assay. This assay included a group of 31 proteins including the Krebs cycle enzymes, housekeeping proteins, an internal standard, and selected other mitochondrial enzymes. The C[160] designates an alkylated cysteine. All analyses are in the positive ion mode with Q1 and Q3 resolution set to 0.7Da. (DOCX) [file pone.0077280.s004.docx]

| Q1 m/z | Q3 m/z | Collision energy (eV) | start time | stop time | peptide sequence | Protein |
| --- | --- | --- | --- | --- | --- | --- |

| 706.39 | 589.36 | 29 | 19.6 | 24.6 | LEEGPPVTTVLTR | Pdha1 |
| --- | --- | --- | --- | --- | --- | --- |
| 706.39 | 690.41 | 29 | 19.6 | 24.6 | LEEGPPVTTVLTR |  |
| 706.39 | 886.53 | 31 | 19.6 | 24.6 | LEEGPPVTTVLTR |  |
| 706.39 | 983.58 | 27 | 19.6 | 24.6 | LEEGPPVTTVLTR |  |
| 706.39 | 1040.60 | 27 | 19.6 | 24.6 | LEEGPPVTTVLTR |  |
| 706.39 | 1169.65 | 27 | 19.6 | 24.6 | LEEGPPVTTVLTR |  |
| 472.28 | 446.27 | 15 | 20.1 | 25.1 | AILAELTGR |  |
| 472.28 | 575.31 | 17 | 20.1 | 25.1 | AILAELTGR |  |
| 472.28 | 646.35 | 15 | 20.1 | 25.1 | AILAELTGR |  |
| 472.28 | 759.43 | 15 | 20.1 | 25.1 | AILAELTGR |  |

| 614.33 | 471.29 | 22 | 15.9 | 20.9 | VLEDNSVPQVK | Pdhb |
| --- | --- | --- | --- | --- | --- | --- |
| 614.33 | 570.36 | 20 | 15.9 | 20.9 | VLEDNSVPQVK |  |
| 614.33 | 657.39 | 20 | 15.9 | 20.9 | VLEDNSVPQVK |  |
| 614.33 | 771.43 | 22 | 15.9 | 20.9 | VLEDNSVPQVK |  |
| 614.33 | 886.46 | 20 | 15.9 | 20.9 | VLEDNSVPQVK |  |
| 614.33 | 1015.50 | 20 | 15.9 | 20.9 | VLEDNSVPQVK |  |
| 666.33 | 402.24 | 23 | 20.5 | 25.5 | EGIEC[160]EVINLR |  |
| 666.33 | 515.33 | 25 | 20.5 | 25.5 | EGIEC[160]EVINLR |  |
| 666.33 | 614.39 | 23 | 20.5 | 25.5 | EGIEC[160]EVINLR |  |
| 666.33 | 743.44 | 25 | 20.5 | 25.5 | EGIEC[160]EVINLR |  |
| 666.33 | 903.47 | 23 | 20.5 | 25.5 | EGIEC[160]EVINLR |  |
| 666.33 | 1032.51 | 27 | 20.5 | 25.5 | EGIEC[160]EVINLR |  |

| 496.25 | 272.16 | 20 | 16.2 | 21.2 | ALSTESVER | Pdk1 |
| --- | --- | --- | --- | --- | --- | --- |
| 496.25 | 403.22 | 20 | 16.2 | 21.2 | ALSTESVER |  |
| 496.25 | 619.30 | 20 | 16.2 | 21.2 | ALSTESVER |  |
| 496.25 | 720.35 | 20 | 16.2 | 21.2 | ALSTESVER |  |
| 759.42 | 674.37 | 23 | 23.3 | 28.3 | AVPLAGFGYGLPISR |  |
| 759.42 | 805.45 | 25 | 23.3 | 28.3 | AVPLAGFGYGLPISR |  |
| 759.42 | 862.47 | 31 | 23.3 | 28.3 | AVPLAGFGYGLPISR |  |
| 759.42 | 1066.56 | 27 | 23.3 | 28.3 | AVPLAGFGYGLPISR |  |
| 759.42 | 1137.60 | 29 | 23.3 | 28.3 | AVPLAGFGYGLPISR |  |

| 485.27 | 243.13 | 20 | 19.0 | 24.0 | EINLLPDR | Pdk2 |
| --- | --- | --- | --- | --- | --- | --- |
| 485.27 | 387.19 | 17 | 19.0 | 24.0 | EINLLPDR |  |
| 485.27 | 727.40 | 23 | 19.0 | 24.0 | EINLLPDR |  |
| 732.90 | 389.25 | 32 | 24.1 | 29.1 | TLSQFTDALVTIR |  |
| 732.90 | 672.44 | 26 | 24.1 | 29.1 | TLSQFTDALVTIR |  |
| 732.90 | 787.46 | 24 | 24.1 | 29.1 | TLSQFTDALVTIR |  |
| 732.90 | 888.51 | 34 | 24.1 | 29.1 | TLSQFTDALVTIR |  |
| 732.90 | 1035.58 | 22 | 24.1 | 29.1 | TLSQFTDALVTIR |  |
| 732.90 | 1250.67 | 24 | 24.1 | 29.1 | TLSQFTDALVTIR |  |

| 599.36 | 327.20 | 23 | 18.0 | 23.0 | LVNTPSVQLVK | Pdk4 |
| --- | --- | --- | --- | --- | --- | --- |
| 599.36 | 770.47 | 19 | 18.0 | 23.0 | LVNTPSVQLVK |  |
| 599.36 | 985.56 | 19 | 18.0 | 23.0 | LVNTPSVQLVK |  |
| 766.91 | 472.28 | 34 | 22.6 | 27.6 | NAPLAGFGYGLPISR |  |
| 766.91 | 642.39 | 30 | 22.6 | 27.6 | NAPLAGFGYGLPISR |  |
| 766.91 | 674.37 | 28 | 22.6 | 27.6 | NAPLAGFGYGLPISR |  |
| 766.91 | 805.45 | 30 | 22.6 | 27.6 | NAPLAGFGYGLPISR |  |
| 766.91 | 862.47 | 24 | 22.6 | 27.6 | NAPLAGFGYGLPISR |  |
| 766.91 | 1066.56 | 26 | 22.6 | 27.6 | NAPLAGFGYGLPISR |  |
